# A study protocol for the development and internal validation of a multivariable prognostic model to determine lower extremity muscle injury risk in elite football (soccer) players, with further exploration of prognostic factors

**DOI:** 10.1186/s41512-019-0063-8

**Published:** 2019-09-19

**Authors:** Tom Hughes, Richard Riley, Jamie C. Sergeant, Michael J. Callaghan

**Affiliations:** 1 Manchester United Football Club, AON Training Complex, Birch Road, Off Isherwood Road, Carrington, Manchester, M31 4BH UK; 2 Arthritis Research UK Centre for Epidemiology, Centre for Musculoskeletal Research, Manchester Academic Health Science Centre, University of Manchester, Manchester, UK; 3 Centre for Prognosis Research, Research Institute for Primary Care and Health Sciences, Keele University, Staffordshire, UK; 4 Centre for Biostatistics, Manchester Academic Health Science Centre, University of Manchester, Manchester, UK; 5 Department of Health Professions, Manchester Metropolitan University, Manchester, UK

**Keywords:** Athlete, Injury prevention, Muscle strain, Prediction, Prognosis, Screening, Sport, Sprains and strains

## Abstract

**Background:**

Indirect muscle injuries (IMIs) are a considerable burden to elite football (soccer) teams, and prevention of these injuries offers many benefits. Preseason medical, musculoskeletal and performance screening (termed periodic health examination (PHE)) can be used to help determine players at risk of injuries such as IMIs, where identification of PHE-derived prognostic factors (PF) may inform IMI prevention strategies. Furthermore, using several PFs in combination within a multivariable prognostic model may allow individualised IMI risk estimation and specific targeting of prevention strategies, based upon an individual’s PF profile. No such models have been developed in elite football and the current IMI prognostic factor evidence is limited. This study aims to (1) develop and internally validate a prognostic model for individualised IMI risk prediction within a season in elite footballers, using the extent of the prognostic evidence and clinical reasoning; and (2) explore potential PHE-derived PFs associated with IMI outcomes in elite footballers, using available PHE data from a professional team.

**Methods:**

This is a protocol for a retrospective cohort study. PHE and injury data were routinely collected over 5 seasons (1 July 2013 to 19 May 2018), from a population of elite male players aged 16–40 years old. Of 60 candidate PFs, 15 were excluded. Twelve variables (derived from 10 PFs) will be included in model development that were identified from a systematic review, missing data assessment, measurement reliability evaluation and clinical reasoning. A full multivariable logistic regression model will be fitted, to ensure adjustment before backward elimination. The performance and internal validation of the model will be assessed. The remaining 35 candidate PFs are eligible for further exploration, using univariable logistic regression to obtain unadjusted risk estimates. Exploratory PFs will also be incorporated into multivariable logistic regression models to determine risk estimates whilst adjusting for age, height and body weight.

**Discussion:**

This study will offer insights into clinical usefulness of a model to predict IMI risk in elite football and highlight the practicalities of model development in this setting. Further exploration may identify other relevant PFs for future confirmatory studies and model updating, or influence future injury prevention research.

**Electronic supplementary material:**

The online version of this article (10.1186/s41512-019-0063-8) contains supplementary material, which is available to authorized users.

## Background

Indirect muscle injuries (IMIs) are the most common injury type in elite football (soccer), predominantly affecting lower extremity muscle groups [1, 2]. Such injuries occur in the absence of direct impact-related trauma (during sprinting for example) [3, 4] and are subclassified into functional disorders without macroscopic structural tissue muscle damage, or structural injuries with clear evidence of muscle disruption [3, 4].

IMIs are problematic for elite teams in terms of both incidence and severity [5], accounting for 30.3% to 47.9% of all injuries that result in time lost to both training and competition [1, 6–9], with the mean and median absence duration reported as 14.4 [1] and 15 days respectively [8]. Player availability is crucial to team prosperity, with vast commercial and financial rewards on offer to successful teams and players [10, 11]. Conversely, player absences through injury negatively affect team performance [12, 13], increase demand on medical services and carry a significant financial burden. As an illustration, for each first team player missing through injury, the daily cost to a participating team in the UEFA Champions League is approximately €17,000 to €20,000 [14, 15].

Periodic health examination (PHE) is used by 94% of elite teams and typically consists of medical examination, musculoskeletal assessment, functional movement evaluation and performance tests, conducted during preseason and in-season periods [16]. PHE is considered important because its intended purposes are to: (1) allow regular health monitoring for underlying but asymptomatic pathology [17]; (2) establish baseline measures for setting rehabilitation or training targets [18]; and (3) identify individuals who are susceptible to common or severe injury types (such as IMIs) [19]. For the latter function, PHE cannot detect causes of injury, but can highlight factors that may be associated with an injury outcome (prognostic factors) and therefore help explain differences in injury risk across individuals within the team [18]. Several prognostic factors could also be used in combination within a multivariable prognostic model to predict an individual’s absolute injury risk [20, 21]. Importantly, both prognostic models and prognostic factors (PFs) can be used to inform management approaches designed to modify an individual’s absolute risk [21]. Despite the potential benefits of prognostic models for shaping injury prevention strategies aimed at clinically important injuries such as IMIs, none have been developed in elite football [22]. In addition, there are significant methodological limitations in the evidence base relating to PHE-derived PFs [22].

Therefore, this study will consist of two primary objectives: (1) to develop and internally validate a prognostic model for individualised IMI risk prediction during a season in elite footballers, using a small number of PHE-derived candidate PFs selected from a previous systematic review [22] and clinical reasoning; and (2) to explore potential PFs associated with IMI outcomes during a season in this elite cohort, using available PHE data from a professional team.

## Methods

### Study design

This study will be of retrospective cohort design, using a population of elite male football players aged 16–40 years old who were employed on a full-time basis at an English Premier League club. The first objective will be conducted in accordance with existing guidelines for model development and internal validation [23, 24] and reported in accordance with the Transparent Reporting of a Multivariable Prediction Model for Individual Prognosis or Diagnosis (TRIPOD) statement [25, 26]. The second objective will be conducted in accordance with existing guidelines [27] and reported in accordance with the REporting recommendations for MARKer prognostic studies [28, 29].

### Data sources

This study will use routinely collected data that was obtained over five seasons (from 1 July 2013 to 19 May 2018). Data collected from the musculoskeletal and performance test components of the club’s PHE will be used to identify candidate PFs. Injury outcome data will also be used to establish the available number of IMI outcomes.

#### Preseason PHE data collection

Each new season commenced from July 1st. Available players completed a mandatory PHE on one of 3 days during the first week of the season. Typically, the musculoskeletal and performance components of the PHE included the following: (1) anthropometric measurements; (2) medical history review (i.e. previous injury history); (3) musculoskeletal examination tests; (4) functional movement and balance tests; and (5) strength and power tests. Detailed descriptions of all tests are provided in Additional file 1.

The PHE test order was self-selected by each player. A standardised warm up was not implemented, although players could undertake their own warm up procedures if they wished. Each component of the PHE test battery was standardised according to a written protocol and conducted by physiotherapists, sports scientists or club medical doctors. To avoid inter-tester variability, the same examiners performed the same test every season and throughout the 5-year data collection period, no examiner attrition occurred.

If a participant was injured at the time of PHE, a risk assessment was completed by medical staff. In such instances, participants only completed tests that were deemed appropriate and safe for the participant’s condition; examiners were therefore not blinded to the injury status of participants.

#### Participant follow-up and injury data collection

Participants were followed up to the last day of each competitive domestic season (defined as the date of the last first team game of the season) irrespective of whether they had completed the PHE procedure or not. Participants completed their routine training and match programmes throughout. For every player in the squad, any injuries that occurred during the season were assessed and electronically documented within 24 h by a club medical doctor or physiotherapist in accordance with the Consensus Statement on Injury Definitions and Data Collection Procedures in Studies of Football Injuries [30]. Musculoskeletal assessments were dependent on the clinical presentation, although typically consisted of observation, effusion, range of movement, muscle length and resisted muscle tests, palpation and special diagnostic manual tests. Radiological imaging was used to assist diagnosis as required. Ultrasound scans were performed by the club medical doctor using a Toshiba Aplio 500 or 1900 machine (Toshiba Corporation, Tokyo, Japan). Magnetic resonance imaging (MRI) was performed as appropriate, using a Canon Vantage Titan 3 T Scanner (Canon Medical Systems, Otowara, Japan) according to sequences determined by the club medical doctor. Images were evaluated by a club medical doctor and an independent musculoskeletal radiologist.

The medical professionals were not blinded to PHE data at the time of diagnosis. These data were not routinely used to inform diagnoses, but instead used to identify functional rehabilitation targets and for benchmarking purposes. Following injury, players completed a rehabilitation programme as directed by club medical staff to enable them to return to training and match participation.

### Participants and eligibility criteria

Eligible participants were identified from a review of the PHE database entries during the dates stated above. During any season, participants were eligible for inclusion into the analysis if they: (1) had an outfield position (i.e. not a goalkeeper); and (2) participated in PHE testing for the relevant season. Participants were excluded from the analysis for any season if they were a triallist player or not contracted to the club at the time of PHE.

### Ethics and data use

Because all data were captured from the mandatory PHE procedure completed through the participants’ employment, informed consent was not required. The anonymity and rights of all participants were protected. The football club granted permission to use these data, and the use of the data for this study was approved by the Research Ethics service at the University of Manchester. This study has been registered on ClinicalTrials.gov, with registered number as NCT03782389.

### Data extraction

All PHE records from eligible participants were extracted and placed into a separate database. Using the club’s electronic medical records system, a further database was generated of all recorded injuries for each season and a manual review of each eligible participant’s medical record was undertaken to ensure accuracy. Each injury was categorised according to the following: (1) contact or non-contact mechanism of injury; (2) injured side; (3) affected body area; (4) injury type, i.e. IMI/ligament/tendon/cartilage/contusion or laceration/bone/concussion/other musculoskeletal injury; and (5) muscle group and diagnostic classification if recorded as an IMI. This process allowed an in-house audit of injury incidence and absolute risk evaluation for each injury type for the squad overall and for those who underwent PHE. All IMIs were then extracted and merged with the PHE database of included participants, for each season in which they remained eligible.

### Outcome measures

For this study, the primary outcome measure will be the occurrence of an initial (index) lower extremity IMI sustained by a participant during a season. Only time-loss injuries will be included; that is, any index lower extremity IMI that occurred during match play or training that resulted in the player being unable to take full part in future match play or training [30]. An IMI was confirmed during the injury assessment procedure outlined above and graded by the club medical doctor or physiotherapist according to the Munich Consensus Statement for the Classification of Muscle Injuries in Sport [4]. This diagnostic classification system was the primary method of muscle injury classification used by the club and has been validated previously [31].

Each participant-season will be treated as independent. If an index lower extremity IMI occurred, the participant’s outcome for the season will be determined and that participant will no longer be considered at risk beyond the time of IMI occurrence. In these circumstances, participants will be included for further analysis at the start of the consecutive season, providing they remain eligible. If participants sustained any upper limb IMI, trunk IMI or non-IMI injury type, these will be ignored and the participant will still be considered at risk of a lower extremity index IMI.

Eligible participants who were loaned out or transferred to another club throughout that season, but had not sustained an index IMI prior to the loan or transfer, will still be considered in the risk set. Participants who sustained an index IMI whilst on loan will be included for analysis, as outlined above. Any participants who were permanently transferred during a season (but had not sustained an index IMI prior to the transfer) will be recorded as not having an IMI event during the relevant season, and they will exit the cohort at this point. A sensitivity analysis may be conducted to evaluate the effect of player loans or transfers on the results.

### Sample size

To maximise statistical power, we have elected to use all data from the 5-season period. This approach agrees with methodological recommendations that data splitting should be avoided, and all available data should be used for model validation [32]. The extracted injury data were audited in parallel with the development of this protocol to determine the number of available index IMI events in the dataset. This was essential to allow calculation of the maximum number of candidate PFs that could be included in model development in order to limit the effects of statistical overfitting [33].

The number of candidate PFs for inclusion in model development will be restricted to a minimum of 10 events per variable (EPV), which is recommended to reduce overfitting and optimism during the development of a logistic regression model [34]. Note that ‘variable’ here means a parameter included (or considered for inclusion) in the model that corresponds to one of the PFs.

Following the audit, the number of independent participant-seasons that will be included for analysis is 317, with 138 index IMI events recorded during the 5-season period. Therefore, we have chosen to restrict the number of parameters (variables) for inclusion in model development to 12, which corresponds to having >10 EPV and thus above the minimum recommendation of 10. We also checked if this met the criteria to minimise overfitting recently proposed by Riley et al. [33]. Assuming the model will have a modest Nagelkerke *R*-squared of 25%, then with an outcome proportion of 0.435, our 12 candidate PF variables correspond to targeting an approximate shrinkage factor of 0.85, and thus a relatively small amount of overfitting (15%) [33]. We deemed this a suitable compromise between increasing the number of PF parameters and minimising the overfitting.

### Candidate prognostic factors

The extracted PHE data were audited as per current methodological recommendations [23], to establish data quality and quantify missing values. This process was also conducted in parallel with the development of this protocol, to assist selection of candidate PFs to be included in either model development or exploration a priori and to inform strategies for handling missing data in the final analysis.

A complete list of all 60 candidate PFs extracted from the PHE dataset is presented in Table 1, with quantitative analysis of missing values for each PF.

**Table 1:** List of candidate prognostic factors, methods and units of measurement, frequency of complete and incomplete observations and proportion of missing observations

Type of prognostic factor	Candidate prognostic factor	Measurement method	Measurement unit	Data type	Complete obs	Missing obs	Percent missing (%)
Anthropometric	Age	Birthdate	Years	Cont.	317	0	0
	Height	Standing height	cm	Cont.	299	18	5.68
	Weight	Digital scales	kg	Cont.	299	18	5.68
	BMI	Height/weight	kg/m^2^	Cont.	294	23	7.26
	Body fat	Skin callipers	%	Cont.	75	242	76.34
Medical history	Frequency of previous IMIs within 3 years prior to PHE	Medical records	Freq.	Dis./cont.	317	0	0
	Most recent previous IMI within 3 years prior to PHE	Medical records	Never, < 6 months, 6–12 months, > 12 months	Cat.	317	0	0
	Frequency of previous foot or ankle injuries within 3 years prior to PHE	Medical records	Freq.	Dis./cont.	317	0	0
	Most recent previous foot or ankle injury within 3 years prior to PHE	Medical records	Never, < 6 months, 6–12 months, > 12 months	Cat.	317	0	0
	Frequency of previous hip or groin injuries within 3 years prior to PHE	Medical records	Freq.	Dis./cont.	317	0	0
	Most recent previous hip or groin injury within 3 years prior to PHE	Medical records	Never, < 6 months, 6–12 months, > 12 months	Cat.	317	0	0
	Frequency of previous knee injuries within 3 years prior to PHE	Medical records	Freq.	Dis./cont.	317	0	0
	Most recent previous knee injury within 3 years prior to PHE	Medical records	Never, < 6 months, 6–12 months, > 12 months	Cat.	317	0	0
	Frequency of previous shoulder injuries within 3 years prior to PHE	Medical records	Freq.	Dis./cont.	317	0	0
	Most recent previous shoulder injury within 3 years prior to PHE	Medical records	Never, < 6 months, 6–12 months, > 12 months	Cat.	317	0	0
	Frequency of previous lumbar spine injuries within 3 years prior to PHE	Medical records	Freq.	Dis./cont.	317	0	0
	Most recent previous lumbar spine injury within 3 years prior to PHE	Medical records	Never, < 6 months, 6–12 months, > 12 months	Cat.	317	0	0
	Frequency of previous iliopsoas IMIs within 3 years prior to PHE	Medical records	Freq.	Dis./cont.	317	0	0
	Most recent previous iliopsoas IMI within 3 years prior to PHE	Medical records	Never, < 6 months, 6–12 months, > 12 months	Cat.	317	0	0
	Frequency of previous adductor IMIs within 3 years prior to PHE	Medical records	Freq.	Dis./cont.	317	0	0
	Most recent previous adductor IMI within 3 years prior to PHE	Medical records	Never, < 6 months, 6–12 months, > 12 months	Cat.	317	0	0
	Frequency of previous hamstring IMIs within 3 years prior to PHE	Medical records	Freq.	Dis./cont.	317	0	0
	Most recent previous hamstring IMI within 3 years prior to PHE	Medical records	Never, < 6 months, 6–12 months, > 12 months	Cat.	317	0	0
	Frequency of previous quadriceps IMIs within 3 years prior to PHE	Medical records	Freq.	Dis./cont.	317	0	0
	Most recent previous quadriceps IMI within 3 years prior to PHE	Medical records	Never, < 6 months, 6–12 months, > 12 months	Cat.	317	0	0
	Frequency of previous calf IMIs within 3 years prior to PHE	Medical records	Freq.	Dis./cont.	317	0	0
	Most recent previous calf IMI within 3 years prior to PHE	Medical records	Never, < 6 months, 6–12 months, > 12 months	Cat.	317	0	0
Musculoskeletal	PROM R hip joint internal rotation	Digital inclinometer	Degrees	Cont.	297	20	6.31
	PROM L hip joint internal rotation	Digital inclinometer	Degrees	Cont.	297	20	6.31
	PROM R hip joint external rotation	Digital inclinometer	Degrees	Cont.	297	20	6.31
	PROM L hip joint external rotation	Digital inclinometer	Degrees	Cont.	297	20	6.31
	R hip flexor muscle length	Digital inclinometer—Thomas test	Degrees	Cont.	294	23	7.26
	L hip flexor muscle length	Digital inclinometer—Thomas test	Degrees	Cont.	294	23	7.26
	R hamstring muscle length /neural mobility	Digital inclinometer—SLR	Degrees	Cont.	297	20	6.31
	L hamstring muscle length /neural mobility	Digital inclinometer—SLR	Degrees	Cont.	297	20	6.31
	R quadriceps muscle length	Goniometer—Ely’s test	Degrees	Cont.	297	20	6.31
	L quadriceps muscle length	Goniometer—Ely’s test	Degrees	Cont.	297	20	6.31
	R calf muscle length	Digital inclinometer—WBL	Degrees	Cont.	297	20	6.31
	L calf muscle length	Digital inclinometer—WBL	Degrees	Cont.	297	20	6.31
	Toe touch in standing	Fingertip-floor distance	cm	Cont.	250	67	21.14
	Sacroiliac joint kinematic function	Gillet’s test	Subjective kinematic function	Cat.	250	67	21.14
Functional movement/balance	Y Balance Test—R anterior translation	Y Balance Test	cm	Cont.	179	138	43.53
	Y Balance Test—L anterior translation	Y Balance Test	cm	Cont.	179	138	43.53
	Y Balance Test—R posteromedial translation	Y Balance Test	cm	Cont.	179	138	43.53
	Y Balance Test—L posteromedial translation	Y Balance Test	cm	Cont.	179	138	43.53
	Y Balance Test—R posterolateral translation	Y Balance Test	cm	Cont.	179	138	43.53
	Y Balance Test—L posterolateral translation	Y Balance Test	cm	Cont.	179	138	43.53
	R relative tibial angles	SLS measured with IMU	Degrees	Cont.	*	*	*
	L relative tibial angles	SLS measured with IMU	Degrees	Cont.	*	*	*
Strength/power	R upper body peak power	Horizontal press	W/kg^−0.67^	Cont.	178	139	43.85
	L upper body peak power	Horizontal press	W/kg^−0.67^	Cont.	178	139	43.85
	R maximal loaded leg extension power	Double leg press	W/kg^−0.67^	Cont.	276	41	12.93
	L maximal loaded leg extension power	Double leg press	W/kg^−0.67^	Cont.	276	41	12.93
	R maximal loaded leg extension velocity	Double leg press	m s^−1^	Cont.	276	41	12.93
	L maximal loaded leg extension velocity	Double leg press	m s^−1^	Cont.	276	41	12.93
	R maximal loaded leg extension force	Double leg press	N/kg^−0.67^	Cont.	276	41	12.93
	L maximal loaded leg extension force	Double leg press	N/kg^−0.67^	Cont.	276	41	12.93
	CMJ height	CMJ	cm	Cont.	275	42	13.25
	CMJ force per kilogram of body mass	CMJ	N/kg	Cont.	275	42	13.25
	CMJ peak power	CMJ	W	Cont.	275	42	13.25

*Freq*. frequency, *obs* observations, *PHE* periodic health examination, *IMI* indirect muscle injury, *WBL* weight bearing lunge, *CMJ* countermovement jump, *PROM* passive range of movement, *SLR* straight leg raise, *SLS* single leg squat, *IMU* inertial measurement units, *BMI* body mass index, *kg/m*^*2*^ kilograms/body height (metres) squared, *W* watts (note: W/kg^−0.67^ has a scaling factor to normalise power to body mass), *N* newtons (note: N/kg^−0.67^ has a scaling factor to normalise force to body mass), *cm* centimetres, *kg* kilograms, *Cont.* continuous, *dis./cont.* discrete treated as continuous, *cat* categorical, *R* right, *L* left, *m s*^*-1*^ metres/second, “–” not applicable/not available, “*” missing data analysis not completed—test evaluated through a reliability/agreement study published as a related part of this project and excluded from further analysis based on the results

#### Missing data

As presented in Table 1, all medical history and age factors were complete (23 factors). Of the 37 remaining candidate PFs, the proportion of missingness ranged from 5.68% (for height and weight) to 76.34% (for body fat). Eleven of these had > 15% missing observations (which included body fat, toe touch in standing, sacroiliac kinematic function, all Y Balance Test and upper body peak power variables). For these factors, the large degree of missingness was because of procedural changes in the PHE process, which meant that these tests were not conducted across all seasons.

For candidate PFs with < 15% missing observations, all tests were conducted consistently across all 5 seasons. For these factors, the sample characteristics of cases with complete PF data were compared to incomplete cases which had at least one missing observation (Table 2).

**Table 2:** Characteristics of cases with complete candidate prognostic factor data, and cases with at least one missing observation for any candidate prognostic factor in the PHE dataset with < 15% missing values

	Complete cases (*n*=264 person-seasons)		Incomplete cases (*n*=53 person-seasons)	
Sample characteristic	Number of obs	Mean (SD)	Number of obs	Mean (SD)
Age (years)	264	20.83 (4.42)	53	23.55 (4.48)
Height (cm)	264	180.58 (6.34)	35	181.12 (6.68)
Weight (kg)	264	74.15 (7.55)	35	77.86 (7.98)
BMI (kg/m^2^ )	264	22.72 (1.76)	30	23.75 (2.24)

*PF* prognostic factor, *obs* observations, *SD* standard deviation, *cm* centimetres, *kg* kilograms, *kg/m*^*2*^ kilograms divided by body height (metres) squared

For complete cases, the mean values of all characteristics were less than incomplete cases, with the largest differences observed in age (20.83 and 23.55 years, respectively) and weight (74.15 and 77.86 kg, respectively). Therefore, a complete case only analysis was not appropriate and we will rather assume that the mechanism of missingness can be considered as missing at random (MAR), where the distribution of missing values is related to values of observed variables [26], to allow imputation and so inclusion of individuals with missing data.

#### Model development and internal validation

We have chosen to conduct the model development before the PF exploration because of the restrictions on the number of PFs permitted to limit potential overfitting of the model.

Because only 12 PF variables will be used in model building, we have defined these candidate PFs a priori (Table 3). Three candidate PFs have known importance based on the results of our previous systematic review so were selected for inclusion [22]. All other PFs listed in Table 1 were eligible unless there were > 15% missing observations or if reliability (where applicable) was classed as fair to poor (ICC < 0.70) [35]. In these cases, the relevant candidate PFs were excluded (Table 4). This was to ensure that only the highest quality data will be used in the analysis, with PFs that would generally be available and routinely measured.

**Table 3:** Restricted set of candidate prognostic factors for model development and validation

Selection method	Candidate prognostic factor	Composite PF	Measurement unit	Number of parameters in model	Measurement method	Data type	Reliability (if applicable)
Systematic review	Age	No	Years	1	Date of birth	Continuous	-
	Frequency of previous IMIs within 3 years prior to PHE	No	Freq.	1	Medical records	Discrete (treated as continuous)	-
	Most recent previous IMI within 3 years prior to PHE	No	< 6 months, 6–12 months, > 12 months	3	Medical records	Categorical	-
Clinical reasoning/data quality	CMJ peak power	No	Watts	1	CMJ using force plates	Continuous	Test-retest ICC = 0.92–0.98 [36]
	PROM hip joint internal rotation difference*	Yes	Degrees	1	Digital inclinometer	Continuous	Intra-rater ICC = 0.90 [37]
	PROM hip joint external rotation difference*	Yes	Degrees	1	Digital inclinometer	Continuous	Intra-rater ICC = 0.90 [37]
	Hip flexor muscle length difference*	Yes	Degrees	1	Digital inclinometer - Thomas test	Continuous	Inter-rater ICC = 0.89 [38]
	Hamstring muscle length /neural mobility difference*	Yes	Degrees	1	Digital inclinometer - SLR	Continuous	Intra-rater ICC =0.95–0.98 [39] Interrater ICC = 0.80–0.97 [40]
	Calf muscle length difference*	Yes	Degrees	1	Digital inclinometer - WBL	Continuous	Inter-rater ICC = 0.80–0.95 [41, 42] Intra-rater = ICC 0.88 [42]
	BMI	Yes	kg/m^2^	1	Composite height (cm) and weight (kg)	Continuous	–

*PF* prognostic factor, *PHE* periodic health examination, *IMI* indirect muscle injury, *WBL* weight bearing lunge, *CMJ* countermovement jump, *PROM* passive range of movement, *ICC* intraclass correlation coefficient, *SLR* straight leg raise, *BMI* body mass index, *kg* kilos, *freq.* frequency, *kg/m*^*2*^ kilograms/body height (metres) squared. "*" denotes between limb differences

**Table 4:** Candidate prognostic factors excluded from both model development and prognostic factor exploration

Type of prognostic factor	Candidate prognostic factor	Composite PF	Measurement unit	Measurement method	Data type	Reason for elimination
Anthropometric	Body fat	No	Percentage	Skin callipers	Continuous	Missing data > 15%
Musculoskeletal	Quadriceps muscle length difference*	Yes	Degrees	Goniometer - Ely's test	Continuous	Intra-rater ICC = 0.69 [43] Inter-rater ICC = 0.66 [43]
	Mean quadriceps muscle length**	Yes	Degrees	Goniometer - Ely's test	Continuous	Intra-rater ICC = 0.69 [43] Inter-rater ICC = 0.66 [43]
	Toe touch in standing	No	Centimetres	Fingertip to floor distance	Continuous	Missing data > 15%
	Sacroiliac joint kinematic function	No	Subjective score	Gillet’s test	Categorical	Missing data > 15%
Functional movement/balance	Y Balance Test—anterior translation difference*	Yes	Centimetres	Y Balance Test	Continuous	Missing data > 15%
	Y Balance Test—mean anterior translation**	Yes	Centimetres	Y Balance Test	Continuous	Missing data > 15%
	Y Balance Test—posteromedial translation difference*	Yes	Centimetres	Y Balance Test	Continuous	Missing data > 15%
	Y Balance Test—Mean posteromedial translation**	Yes	Centimetres	Y Balance Test	Continuous	Missing data > 15%
	Y Balance Test—posterolateral translation difference*	Yes	Centimetres	Y Balance Test	Continuous	Missing data > 15%
	Y Balance Test—mean posterolateral translation**	Yes	Centimetres	Y Balance Test	Continuous	Missing data > 15%
	R relative tibial angles	No	Degrees	SLS measured with Dorsavi Viperform IMU	Continuous	Within-session ICCs = 0.27–0.75 Between-session ICCs = 0.55–0.77 [44]
	L relative tibial angles	No	Degrees	SLS measured with Dorsavi Viperform IMU	Continuous	Within-session ICCs = 0.27–0.75 Between-session ICCs = 0.55–0.77 [44]
Strength/power	Upper body peak power difference*	Yes	Normalised watts per kilo (W/kg^−0.67^)	Double horizontal press using a Keiser Chest Press Air 350 machine	Continuous	Missing data > 15%
	Mean upper body peak power**	Yes	Normalised watts per kilo (W/kg^−0.67^)	Double horizontal press using a Keiser Chest Press Air 350 machine	Continuous	Missing data > 15%

*ICC* intraclass correlation coefficient, *SLS* single leg squat, *W* watts, (note that W/kg^−0.67^ has a scaling factor to normalise power to body mass), *kg* kilos, *IMU* inertial measurement units, *R* right, *L* left. “*” denotes between limb differences and “**” denotes the mean of the measurements for both limbs

Co-linearity amongst factors within a logistic regression model can cause inaccuracies in standard error and confidence interval estimates [45], so a scatterplot matrix was used to informally assess between-factor correlations for eligible PFs. If PFs were highly correlated, one of the PFs was dropped or new composite PFs were generated and replaced the original factors (highlighted in Tables 3, 4 and 5). Typically, this occurred where measurements examined both right and left limbs separately; composite factor variables were therefore created for both between-limb measurement differences and the mean of the measurements for both limbs.

**Table 5:** Candidate prognostic factors—exploration

Type of prognostic factor	Candidate Prognostic factor	Composite PF	Measurement unit	Measurement method	Data type	Reliability (if applicable/available)
Anthropometric	Height	No	Centimetres	Standing height measure	Continuous	–
	Weight	No	Kilograms	Digital scales	Continuous	–
Medical history	Frequency of previous foot or ankle injuries within 3 years prior to PHE	No	Freq.	Medical records	Continuous	–
	Most recent previous foot or ankle injury within 3 years prior to PHE	No	< 6 months, 6–12 months, > 12 months	Medical records	Categorical	–
	Frequency of previous hip or groin injuries within 3 years prior to PHE	No	Freq.	Medical records	Continuous	–
	Most recent previous hip or groin injury within 3 years prior to PHE	No	< 6 months, 6–12 months, > 12 months	Medical records	Categorical	–
	Frequency of previous knee injuries within 3 years prior to PHE	No	Freq.	Medical records	Continuous	–
	Most recent previous knee injury within 3 years prior to PHE	No	< 6 months, 6–12 months, > 12 months	Medical records	Categorical	–
	Frequency of previous shoulder injuries within 3 years prior to PHE	No	Freq.	Medical records	Continuous	–
	Most recent previous shoulder injury within 3 years prior to PHE	No	< 6 months, 6–12 months, > 12 months	Medical records	Categorical	–
	Frequency of previous lumbar spine injuries within 3 years prior to PHE	No	Freq.	Medical records	Continuous	–
	Most recent previous lumbar spine injury within 3 years prior to PHE	No	< 6 months, 6–12 months, > 12 months	Medical records	Categorical	–
	Frequency of previous iliopsoas IMIs within 3 years prior to PHE	No	Freq.	Medical records	Continuous	–
	Most recent previous iliopsoas IMI within 3 years prior to PHE	No	< 6 months, 6–12 months, > 12 months	Medical records	Categorical	–
	Frequency of previous adductor IMIs within 3 years prior to PHE	No	Freq.	Medical records	Continuous	–
	Most recent previous adductor IMI within 3 years prior to PHE	No	< 6 months, 6–12 months, > 12 months	Medical records	Categorical	–
	Frequency of previous hamstring IMIs within 3 years prior to PHE	No	Freq.	Medical records	Continuous	–
	Most recent previous hamstring IMI within 3 years prior to PHE	No	< 6 months, 6–12 months, > 12 months	Medical records	Categorical	–
	Frequency of previous quadriceps IMIs within 3 years prior to PHE	No	Freq.	Medical records	Continuous	–
	Most recent previous quadriceps IMI within 3 years prior to PHE	No	< 6 months, 6–12 months, > 12 months	Medical records	Categorical	–
	Frequency of previous calf IMIs within 3 years prior to PHE	No	Freq.	Medical records	Continuous	–
	Most recent previous calf IMI within 3 years prior to PHE	No	< 6 months, 6–12 months, > 12 months	Medical records	Categorical	–
Musculoskeletal	Mean PROM hip joint internal rotation**	Yes	Degrees	Digital inclinometer	Continuous	Intra-rater ICC = 0.90 [37]
	Mean PROM hip joint external rotation**	Yes	Degrees	Digital inclinometer	Continuous	Intra-rater ICC = 0.90 [37]
	Mean hip flexor muscle length**	Yes	Degrees	Digital inclinometer - Thomas test	Continuous	Inter-rater ICC = 0.89 [38]
	Mean hamstring muscle length/neural mobility**	Yes	Degrees	Digital inclinometer - SLR	Continuous	Intra-rater ICC = 0.95–0.98 [40] Inter-rater ICC = 0.80–0.97 [40]
	Mean calf muscle length**	Yes	Degrees	Digital inclinometer - WBL	Continuous	Inter-rater ICC = 0.80–0.95 [41, 42] Intra-rater ICC = 0.88 [42]
Strength/power	Maximal loaded leg extension power difference*	Yes	Normalised watts per kilo (W/kg^−0.67^)	Double leg press test using a Keiser Air 300 machine	Continuous	Test-retest ICC = 0.886 [46]
	Mean of maximal loaded leg extension power**	Yes	Normalised watts per kilo (W/kg^−0.67^)	Double leg press test using a Keiser Air 300 machine	Continuous	Test-retest ICC = 0.886 [46]
	Loaded maximal leg extension velocity difference*	Yes	Peak velocity (m s^−1^)	Double leg press test using a Keiser Air 300 machine	Continuous	Test-retest ICC = 0.792 [46]
	Mean of maximal loaded leg extension velocity**	Yes	Peak velocity (m s^−1^)	Double leg press test using a Keiser Air 300 machine	Continuous	Test-retest ICC = 0.792 [46]
	Loaded maximal leg extension force difference*	Yes	Normalised peak force (N/kg^−0.67^ )	Double leg press test using a Keiser Air 300 machine	Continuous	Test-retest ICC = 0.914 [46]
	Mean of maximal loaded leg extension force**	Yes	Normalised peak force (N/kg^−0.67^)	Double leg press test using a Keiser Air 300 machine	Continuous	Test-retest ICC = 0.914 [46]
	CMJ force per kilogram of body mass	No	Force per kg (N/kg)	CMJ using force plates	Continuous	–
	CMJ height	No	Centimetres	CMJ using force plates	Continuous	Test-retest ICC = 0.80–0.88 [47]

*PF* prognostic factor, *PHE* periodic health examination, *IMI* indirect muscle injury, *WBL* weight bearing lunge, *CMJ* countermovement jump, *PROM* passive range of movement, *ICC* intraclass correlation coefficient, *SLR* straight leg raise, *kg* kilos, *W* watts, (note that W/kg^-0.67^ has a scaling factor to normalise power to body mass), *N* newtons, (note that N/kg^-0.67^ has a scaling factor to normalise force to body mass), *m s*^*−1*^ metres/second,* "-"* not applicable/not available. “*” denotes between limb differences and “**” denotes the mean of the measurements for both limbs

Of the remaining eligible PFs, 9 further candidate factor variables were selected for inclusion, through use of clinical reasoning to identify those with a biologically plausible association with IMI development. The final set of 12 PF variables is shown in Table 3.

#### Prognostic factor exploration

Candidate PFs that were that were not selected for use in model development (but not excluded) will be eligible for further exploratory analysis (Table 5). This will allow identification of other potentially useful associations which may assist future analyses or updating of the model created under the first objective of this investigation.

### Statistical analysis

#### Model development and internal validation

Multivariable logistic regression will be used for the analysis as this is an appropriate method where outcomes are binary [26] and independent variables (PFs) are continuous, categorical or a combination [45]. Initially, we will fit a full multivariable model containing all 12 candidate PF variables to ensure a fully adjusted model prior to the potential elimination of unimportant candidate factors [23]. Backward elimination will then be used to successively remove non-significant factors with *p* values of greater than 0.157. This threshold was set to approximate equivalence with Akaike’s Information Criterion [48]. Using backward elimination in this way may deliver a more parsimonious model which is therefore easier to implement in clinical practice than a full model. Where possible, we will retain continuous candidate PFs in their continuous form to avoid statistical power loss [49].

Because the missing data mechanism is considered as missing at random (MAR), multiple imputation (MI) will be implemented, using 50 imputations. We have chosen to utilise MI because it avoids excluding participants from the analysis, is an effective method of handling missing prognostic factor information and can be used to account for uncertainty in missing data [50].

The apparent performance of the developed model will be summarised in the development datasets (averaged over imputation datasets), via calibration and discrimination. Model calibration determines performance in terms of the agreement between predicted outcome risks and those actually observed [51]. Graphical plots are useful to assess calibration [23], so will be produced and utilised in the analysis. We will calculate calibration-in-the-large (CITL, ideal value of 0), which quantifies the systematic error in model predictions (overall agreement). A related measure is E/O (ideal value of 1), which gives the ratio of the mean of the predicted (expected (E)) risks against the mean of the observed risks (O) [51, 52]. A calibration slope will also be calculated, where a value of 1 equals perfect calibration [26]. Models demonstrate perfect calibration within development data, but in new data, the slope may be < 1 due to overfitting in the model development dataset (see below for how this will be handled) [52].

Discrimination performance is a measure of a model’s ability to separate participants who have experienced an outcome compared to those who have not, quantified using the *C* (concordance) statistic (equivalent to the area under the ROC curve) [23]. This index measure will be calculated for the development model, where 1 demonstrates perfect discrimination, whilst 0.5 indicates that discrimination is no better than by chance alone.

To quantify the degree of optimism due to overfitting, our model will be internally validated using bootstrap re-sampling. This will be conducted as previously outlined [26, 53]. The prognostic factor variable selection procedure and model construction will be repeated for 200 bootstrap samples. For each sample, the difference in bootstrap apparent performance (of the bootstrap model in the bootstrap data) and test performance (of the bootstrap model in the original dataset) will be averaged across the 200 samples, to obtain a single estimate of optimism for each performance statistic. Then, to calculate optimism-adjusted estimates of performance for our new model, the estimates of optimism will be subtracted from the original apparent estimates of performance.

The optimism-adjusted calibration slope will provide a uniform shrinkage factor, which will be applied to all prognostic factor effects in the developed model to adjust (shrink) for overfitting. The intercept of the model will then be re-estimated accordingly. This will then form our final model.

#### Prognostic factor exploration

All remaining candidate factors that are eligible for exploration (Table 5) will undergo univariable logistic regression analyses to determine unadjusted associations with IMIs. Candidate PFs will also be incorporated into multivariable logistic regression models to determine odds ratios after adjustment for age, height and body weight. Note that because age was included as a candidate in the original model and will also be used for adjustment purposes in the exploratory multivariable models, the total number of candidate PFs eligible for exploration is 36. Exploration of non-linear associations between candidate factors and index IMI outcomes will also be evaluated using a fractional polynomial approach [49].

## Discussion

Although previous studies in elite football have investigated the association between factors obtained during PHE and IMIs using multivariable models, none have developed, validated or evaluated the performance a prognostic model for injury prediction purposes [22]. Whilst it is possible to develop a prognostic model from PHE data [18], our investigation will offer valuable insights into the practical aspects of this process and the clinical usefulness of a model when applied to an individual football club. Our findings may also outline how these principles may be used in future at other clubs or sports, or on larger datasets which could be derived from several collaborating clubs.

Despite the availability of high-quality PHE and injury data, the relatively small number of outcomes in this dataset is problematic and will permit only a limited selection of candidate prognostic factors for use in model development. Utilising more than one prognostic factor variable for every 10 injury outcomes may cause significant issues with model overfitting, where spurious observed relationships occur because of regression value distortion [34]. This leads to an overestimation of predictive performance (optimism) which is especially evident in small datasets [54]. To limit the effects of overfitting, only 10 PFs (resulting in 12 variables) will be permitted and use of data reduction methods have been required to select appropriate candidate factors for inclusion.

PFs for clinical injury outcomes are either intrinsic (person specific) or extrinsic (environment specific) [55] and can be modifiable or non-modifiable [56]. Only the non-modifiable factors of increasing age and history of previous muscle injury have been shown to have modest prognostic value for hamstring muscle injuries in elite footballers [22], so will be included in model development. However, their non-modifiable nature means that they have limited use in terms of informing injury prevention strategies. To enhance the clinical applicability of the model, other potentially relevant and modifiable factors have been selected for inclusion.

The methodological shortcomings in the literature mean that only three candidate prognostic factors could be selected for model development from our previous systematic review [22]. Subsequently, candidate PF selection for our model has been largely based upon the evaluation of collinearity, measurement reliability and clinical reasoning, which means that it is possible that some important factors have not been considered. It is also possible that some potentially useful factors have been excluded on the basis of having >15% of missing values. As such, only modest performance of this initial model is expected.

It is acknowledged that the proposed prognostic model will assume that participants are independent for each season and utilise the binary outcome of at least one IMI in a season, rather than evaluating time to individual IMI events. This means that we will not account for within-person correlations from season to season. Although this is not fully representative of the real world, because this is a novel area and we are restricted to a relatively small dataset, we have elected to perform the analyses in a more simplistic manner in the first instance. Further, more complex analyses may be conducted in the future.

To assess the generalisability of a prognostic model, it should be externally validated using data from another location [21, 24], such as a dataset from another comparable elite level football team. Because there is likely to be considerable between-team heterogeneity in PHE processes [16], candidate prognostic factors within our model may not translate externally at this time. There are no immediate plans to externally validate this model. However, depending on the outcome of the model development and exploratory objectives, it may be possible to conduct a future prospective temporal validation study within the same football club, or external validation study in different population. If feasible, such investigations will require a separate associated protocol.

The current evidence relating to PFs for injury in football is frequently flawed due to issues with the reliability of data measurement, adjustment, dichotomisation and potential diagnostic misclassification, so there is a need for further studies that address these issues [22]. Further hypothesis-free exploratory studies that investigate many factors (including those that are not necessarily biologically plausible) may assist with identification of new factors that may help inform management decisions and monitoring purposes [20]. Furthermore, these types of studies are helpful because new PFs may be used to update a developed model to improve performance [57]. We have therefore outlined an exploratory objective to investigate the association between IMIs and other factors from the current dataset, using a validated diagnostic outcome classification system and recommended statistical approaches, ensuring that where possible, analysis of continuous data remains on the continuous scale to explore linear and non-linear associations.

We anticipate that this investigation will provide a comprehensive evaluation of what is currently possible in terms of using PHE data to predict IMIs at an elite football club, by adhering to transparent reporting procedures and current best practice for model development, validation and exploration of potential PFs. We hope this study will also identify further research priorities for this novel and potentially valuable area of sports/football medicine research.

## Additional file

Additional file 1: Detailed descriptions of all PHE tests. (DOCX 46 kb)

## Data Availability

An anonymised summary of the dataset that will be generated and analysed during this current study may be available from the corresponding author on reasonable request.
