# Supplementary material for: A study protocol for the development and internal validation of a multivariable prognostic model to determine lower extremity muscle injury risk in elite football (soccer) players, with further exploration of prognostic factors
Source: Diagn Progn Res. 2019 Sep 19;3:19. doi: 10.1186/s41512-019-0063-8 (PMC6751574; doi:10.1186/s41512-019-0063-8)
Supplement: Supplementary file 1 — Detailed descriptions of all PHE tests. (DOCX 46 kb) [file 41512_2019_63_MOESM1_ESM.docx]

**A Study Protocol for the Development and Internal Validation of a Multivariable Prognostic Model to Determine Lower Extremity Muscle Injury Risk in Elite Football (Soccer) Players, with Further Exploration of Prognostic Factors. (Hughes et al (2019))**

**Additional File 1 – Detailed descriptions of all PHE tests.**

1. ***Anthropometric measurements***

For each participant, height was recorded in centimetres (cm) and body weight was measured in kilograms (kg) using a combined weight and height scales (Seca, Hamburg, Germany). Body mass index was calculated as weight (kg) divided by the squared height (metres). Body compositions were estimated through percentage body fat calculations; using a generic skinfold calliper, measurements were taken from the of triceps, subscapular, lower abdominal, anterior thigh, pectoral and iliac crest anatomical locations by the same examiner.

1. ***Past Medical/Injury history***

All participants underwent an interview with a club medical doctor to discuss their past medical and injury history. For participants who were transferred to the club during the data collection period, their initial PHE included questions regarding family medical history and any previous significant injuries sustained at their previous club (which resulted in time lost to training of > 7-10 days). This information was cross referenced with their medical records received from the previous football club. Subsequent PHEs consisted of an interview which reviewed the medical information and injury history that was routinely recorded on the medical notes system during each participant’s employment with the club.

####

#### *Musculoskeletal examination*

Participants underwent a series of musculoskeletal tests that were completed by one examiner, while another examiner monitored the procedure for any accessory or compensatory movements and verified the measurement before recording it into a database. If any accessory or compensatory movements were noted the procedure for that particular test was repeated. All examination procedures were performed according to a standardised protocol.

####

#### *Passive Hip Internal/External Rotation*

Hip rotational range of movement was used to evaluate hip joint mobility.(1) Participants laid supine with both thighs supported by the treatment plinth and knees flexed over the end of the plinth to allow the lower legs to hang freely. A digital inclinometer (Digi-Pas Uk – Tarax Technology Ltd, Cambridge) was placed on the medial aspect of the tibia immediately proximal to the medial malleolus of the tibia. The examiner instructed the participant to flex the contralateral hip and knee and place the foot on the plinth to stabilise the pelvis. The hip was passively rotated to the maximal range of external rotation, until resistance prevented any further movement or symptoms were experienced. The range was documented in degrees and entered into the database. The test was repeated for passive internal rotation. Both procedures were repeated on the contralateral limb. Use of a digital inclinometer to evaluate hip rotation has previously been shown to have excellent intra-rater (intraclass correlation coefficient (ICC)= 0.84- 0.90)(1, 2), inter-rater (ICC=0.89-0.93)(3) and test-retest reliability (ICC=0.93-0.96).(4)

#### *Modified Thomas Test*

The modified Thomas test was used to measure the flexibility of the anterior hip musculature.(5) Participants stood against a plinth which was adjusted so it was aligned to the height of the anterior superior iliac spine (ASIS). Participants lay supine on the edge of the plinth with both legs hanging freely. Both hips and knees were maximally flexed and held to the chest with both hands of the participant. One limb was released and the examiner extended the hip with as far as possible, whilst ensuring that the lumbar spine remained in contact with the plinth and the contralateral knee maintained the flexed position. A digital inclinometer (Digi-Pas Uk – Tarax Technology Ltd, Cambridge) was placed on the muscle belly of the upper 1/3 of the quadriceps and the measurement was recorded in degrees. If the femur was angled so it was above horizontal, a negative measurement was recorded. If it was below the horizontal, a positive measure was recorded. The procedure was then repeated on the contralateral leg. The inter-rater reliability of using an inclinometer with this test has be previously established as good (ICC= 0.89).(6)

#### *Ely’s Test*

Ely’s test was used to measure rectus femoris length and knee joint mobility.(5, 7) Participants laid prone on the treatment plinth. The examiner instructed the participant to flex the hip closest to the edge of the plinth to 90 degrees and place the foot on the floor in order to stabilize the pelvis. The contralateral leg (which rested on the plinth) was then passively flexed at the knee to its maximum range, until soft tissue resistance limited movement or pain or discomfort was experienced. The range of knee flexion was measured with a generic plastic goniometer. This was placed over the lateral epicondyle of the knee, with the stationery arm aligned with the greater trochanter and the moving arm aligned with the lateral malleolus of the fibula. The range was recorded in degrees and the test was repeated on the contralateral limb. Fair intra and inter-rater reliability for this method of measurement has been established previously (ICC 0.69 and 0.66 respectively).(7)

#### *Straight Leg Raise*

The straight leg raise (SLR) was used to measure of hamstring and neurodynamic mobility.(5) The participant laid supine on a plinth. A digital inclinometer (Digi-Pas Uk – Tarax Technology Ltd, Cambridge) was placed on the anterior margin of the tibia (10 centimetres distal to the tibial plateau) by the examiner. The examiner maintained full knee extension and passively flexed the hip to its maximal range in the sagittal plane, or to the point where any symptoms were noted. The range was recorded in degrees and the test was repeated on the contralateral limb. Previously for this method of measurement, intra-rater reliability has been shown to be excellent (ICC 0.95-0.98) (8) and inter-rater reliability has been shown to be good (ICC 0.80-0.97). (9)

####

#### *Weight Bearing Lunge*

The weight bearing lunge (WBL) was used to measure dorsiflexion mobility of the ankle.(10) Participants were instructed to stand along a tape line which was perpendicular to the wall, where the second toe and centre of the heel were placed over the line. A digital inclinometer (Digi-Pas Uk – Tarax Technology Ltd, Cambridge) was placed over the anterior margin of the tibia (10 centimetres distal to the tibial plateau) in a neutral position. Participants were instructed to step as far forward as possible with the contralateral leg, into a forward stride position with both hands on the wall in front, which dorsiflexed the rear ankle. Participants were instructed to lean forward as much as possible until resistance or symptoms were experienced, whilst maintaining knee extension with contact of the heel on the floor. If the heel lifted from the ground the test was deemed as invalid and then repeated. The maximal range of dorsiflexion was documented in degrees. The test was repeated on the contralateral leg. Previously, this method has been shown to have good intra-rater reliability (ICC=0.88) and good to excellent inter-rater reliability (ICC 0.80- 0.95.(11, 12)

#### *Toe Touch in Standing Test*

The toe-touch in standing (TTS) test was used as a measure of global lumbar, pelvic and hamstring flexibility.(13) Participants started in a relaxed standing posture and then instructed to flex forward as far as possible in an attempt to touch their toes. If the participant was unable to reach the floor due to symptoms or resistance, the examiner measured the distance from the floor to the participants most distal fingertip with a generic cloth tape measure, recorded in cm. If the participant was able to touch the floor, a score of 0 cm was given. The TTS has been shown to have good test-retest reliability (ICC 0.89).(14, 15)

*Gillet Test*

The Gillet test was used as a measure of sacroiliac joint kinematic dysfunction.(5) Participants stood in front of the examiner (with their back to them), whom palpated the posterior superior iliac spine and the base of the sacrum. Participants then were instructed to stand on the ipsilateral leg, while flexing the contralateral hip to 90 degrees.(5) The test was repeated on the contralateral side and differences in posterior pelvic (inominate) rotation were compared and subjectively graded as either ‘normal’ (i.e. similar posterior rotation bilaterally), ‘hypomobile’ (i.e. where the inominate of the side tested rotated less than the contralateral side) or ‘hypermobile’ (i.e. where the inominate of the side tested rotated more than the contralateral side).

1. ***Functional Movement and Balance Tests***

#### *Single leg Squat*

The single leg squat (SLS) was used to measure dynamic frontal plane knee alignment during a functional task. (16) The ViPerform inertial measurement unit (IMU) system (Dorsavi, Melbourne, Australia) was applied to the tibia according to the manufacturer’s instructions, using a proprietary leg template to identify the correct site, based on each participant’s height. Disposable application pads (Dorsavi, Melbourne, Australia) were affixed to the medial tibia and the IMU units were clipped into position. From a single leg standing position, participants were instructed to perform a SLS to at least 45° and no greater than 60° knee flexion(17) over 5 seconds using a timer, which was verbalised by an examiner; second 1 initiated the SLS, second 3 indicated the position of maximal knee flexion and second 5 indicated the trial end, where ground contact with both legs was permitted.(18) This standardisation reduced any velocity effects on kinematics. Data were captured for 5 trials per leg, with the left side evaluated before the right.

Relative tibial angles were measured in real time and saved to a computer using the manufacturer’s software (ViPerform 5.10, Dorsavi, Melbourne, Australia), where positive values indicated tibial abduction (proximal tibial lateral displacement from vertical) and negative values indicated tibial adduction (proximal tibial medial displacement from vertical). (16) Our related study demonstrated that the within-session reliability ranged from poor to good (ICC=0.27-0.75), while between-session reliability was found to be fair to good (ICC=0.55-0.77) although was dependent on limb kicking preference. (16)

####

#### *Y Balance Test (YBT)*

The Lower Quarter Y Balance Test was used to measure dynamic balance performance (19) by challenging range of movement, strength and proprioception neuromuscular postural control mechanisms.(20, 21) The test was conducted described previously (22) using the Y Balance Test Kit (Functional Movement Systems, Chatham, USA). The test procedure was explained and demonstrated to each participant. The YBT was completed barefoot; participants placed one foot on the central plastic platform in a single leg standing starting position. The distal border of the great toe touched the starting line on the platform and hands were placed on the hips. The participant was instructed to reach maximally with free leg by pushing the sliding blocks connected to the anterior, posterolateral and posteromedial poles. The final distance was identified by the final block position (recorded in cm) and test movements were completed by returning to the starting single leg stance position. Tests were deemed invalid if the participant was unable to maintain the single leg stance, lifted the heel, moved the foot from the starting position or failed to return to the start posture.(22, 23) Invalid tests were repeated. the test was completed again. Test repetitions were completed in all directions twice using both legs as stance legs, unless a fail was recorded and the test was repeated. The YBT-LQ has been demonstrated to have excellent intra-rater (ICC 0.85-0.91) and interrater reliability (ICC 0.99-1.00).(20)

1. ***Strength and Power Tests***

#### *Maximal Loaded Leg Press*

This test was used to measure the force, power and velocity for both lower limbs during an incremental leg double press test, which progressed from minimal to maximal resistance, using a Keiser Air 300 machine (Keiser UK, Tetbury, Gloucestershire). Prior to this test participants warmed up on a Keiser M3 exercise cycle (Keiser UK, Tetbury, Gloucestershire) for 5 mins at resistance level one. Participants sat on the leg press machine and an appropriate seat position was selected, with the feet firmly rested on the footplates and the knees were at 90 degrees of flexion. Participants were instructed to generate as much force as possible, as quickly as possible by pushing against the footplate and keeping the spine flat against the seat during completion of the test movement. A test was deemed invalid if any compensatory movements were observed (such as elevating the pelvis from the seat to gain a mechanical advantage) and the test was repeated. The test consisted of repeated single repetitions, with each repetition increasing in resistance until the maximum resistance was reached and the participant could not complete the leg press movement. The measurements obtained for this test are peak power (Watts/kg(W/kg)), Peak velocity (metres/second (m/s)) and peak force (Newtons/kg (N/kg) ) for each limb. The test-retest reliability has been shown to have good-excellent reliability for the power (ICC= 0.89), velocity (ICC= 0.79) and force (ICC=0.91) parameters. (24)

#### *Maximal Loaded Horizontal Press*

This test was used to measure the force, power and velocity for both upper limbs during an incremental horizontal double chest press test, which progressed from minimal to maximal resistance, using a Keiser Air 350 machine (Keiser UK, Tetbury, Gloucestershire). An appropriate seat position was selected for each participant. The seat position was adjusted so that the feet were firmly placed on the ground with the spine resting firmly against the backrest. Participants were instructed to generate as much force as possible, as quickly as possible by pushing against the handles. A test was deemed as invalid if any compensatory movements were observed and the test was repeated. The test consisted of repeated single repetitions, with each repetition increasing in resistance until the maximum resistance was reached and the participant could not complete the chest press movement. The measurements obtained for this test are peak power (W/kg), peak velocity (m/s) and peak force (N/kg) for each limb. Despite a literature search, there was no evidence that has specifically investigated the reliability of this measurement method.

####

#### *Countermovement jump (CMJ)*

This test provided a functional measure of explosive power.(25, 26) All CMJs were performed on a dual plate force platform system (ForceDecks FD4000, ForceDecks, Salford, UK) and analysed using the manufacturer’s dedicated software. The test procedure was explained and demonstrated to each participant. Participants were instructed to stand on the platform, with foot placement approximately hip width apart and their hands placed on the pelvis for the duration of the CMJ. In one dynamic movement, participants squatted to approximately 90 degrees of knee flexion before forcefully propelling vertically into the jump phase of the movement through triple extension of hips, knees and ankles, landing approximately in the original start position. Four CMJ repetitions were completed, although if jump height progressively increased with each effort, further CMJs were completed until height scores plateaued. If participants hands shifted position from the pelvis or the legs did not remain in an extended position during the take-off or flight phase then the repetition was deemed invalid and the test was repeated. The measurements taken from this test were peak jump height (cm), peak power (watts) and force per kg of body mass (N/kg). The test-retest reliability of peak jump height has been previously shown to be good (ICC= 0.80-0.88).(27) Similarly, for CMJ power, test-retest reliability has been shown to be excellent (ICC=0.92-0.98)(28).

**References**

1. Roach S, San Juan JG, Suprak DN, Lyda MA. Concurrent validity of digital inclinometer and universal goniometer assessing passive hip mobility in healthy subjects. The International Journal of Sports Physical Therapy. 2013;8(5):680-8.

2. Krause DA, Hollman JH, Krych AJ, Kalisvaart MM, Levy BA. Reliability of hip internal rotation range of motion measurement using a digital inclinometer. Knee Surg Sports Traumatol Arthrosc. 2015;23(9):2562-7.

3. Hollman JH, Ginos BE, Kozuchowski J, Vaughn AS, Krause D, Youdas J. Relationships Between Knee Valgus, Hip-Muscle Strength, and Hip-Muscle Recruitment During a Single-Limb Step-Down. Journal of Sport Rehabilitation. 2009;18:104-17.

4. Pua YH, Wrigley TV, Cowan SM, Bennell KL. Intrarater test-retest reliability of hip range of motion and hip muscle strength measurements in persons with hip osteoarthritis. Arch Phys Med Rehabil. 2008;89(6):1146-54.

5. Magee DJ. Orthopedic Physical Assessment. 5th Ed. ed. Missouri: Saunders Elsevier; 2008.

6. Clapis PA, Davis SM, Davis RO. Reliability of inclinometer and goniometric measurements of hip extension flexibility using the modified Thomas test. Physiotherapy theory and practice. 2008;24(2):135-41.

7. Peeler J, Anderson JE. Reliability of the Ely's test for assessing rectus femoris muscle flexibility and joint range of motion. Journal of orthopaedic research : official publication of the Orthopaedic Research Society. 2008;26(6):793-9.

8. Boyd BS. Measurement properties of a hand-held inclinometer during straight leg raise neurodynamic testing. Physiotherapy. 2012;98(2):174-9.

9. Gabbe BJ, Bennell KL, Wajswelner H, Finch CF. Reliability of common lower extremity musculoskeletal screening tests. Phys Ther Sport. 2004;5(2):90-7.

10. Catalatayud J, Martin F, Gargallo P, Garcia-Redondo J, Colado JC, Marin PJ. The validity and reliability of a new instrumented device for measuring ankle dorsiflexion range of motion. The International Journal of Sports Physical Therapy. 2015;10(2):197-202.

11. Williams CM, Caserta AJ, Haines TP. The TiltMeter app is a novel and accurate measurement tool for the weight bearing lunge test. J Sci Med Sport. 2013;16(5):392-5.

12. Munteanu SE, Strawhorn AB, Landorf KB, Bird AR, Murley GS. A weightbearing technique for the measurement of ankle joint dorsiflexion with the knee extended is reliable. J Sci Med Sport. 2009;12(1):54-9.

13. Bennell K, Tully E, Harvey N. Does the toe-touch test predict hamstring injury in Australian rules footballers? Aust J Physiother. 1999;45(2):103-9.

14. Ayala F, Sainz de Baranda P, De Ste Croix M, Santonja F. Reproducibility and criterion-related validity of the sit and reach test and toe touch test for estimating hamstring flexibility in recreationally active young adults. Phys Ther Sport. 2012;13(4):219-26.

15. Ayala F, Sainz de Baranda P, De Ste Croix M, Santonja F. Absolute reliability of five clinical tests for assessing hamstring flexibility in professional futsal players. J Sci Med Sport. 2012;15(2):142-7.

16. Hughes T, Jones RK, Starbuck C, Picot J, Sergeant JC, Callaghan MJ. Are tibial angles measured with inertial sensors useful surrogates for frontal plane projection angles measured using 2-dimensional video analysis during single leg squat tasks? A reliability and agreement study in elite football (soccer) players. J Electromyogr Kinesiol. 2019;44:21-30.

17. Zeller BL, McCrory JL, Kibler WB, Uhl TL. Differences in Kinematics and Electromyographic Activity Between Men and Women during the Single-Legged Squat. Am J Sports Med. 2003;31(3):449-68.

18. Herrington L. Knee valgus angle during single leg squat and landing in patellofemoral pain patients and controls. Knee. 2014;21(2):514-7.

19. Coughlan GF, Fullam K, Delahunt E, Gissane C, Caulfield BM. A Comparison Between Performance on Selected Directions of the Star Excursion Balance Test and the Y Balance Test. Journal of Athletic Training (Allen Press). 2012;47(4):366-71.

20. Plisky MS, Gorman PP, Butler RJ, Kiesel KB, Underwood F, Elkins B. The reliability of an instrumented device for measuring components of the Star Excursion Balance Test. N Am J Sports Phys Ther. 2009;4(2).

21. Hertel J, Braham RA, Hale SA, Olmsted-Kramer LC. Simplifying the Star Excursion Balance Test: Analyses of subjects With and Without Chronic Ankle Instability. J Orthop Sports Phys Ther. 2006;36:131-7.

22. Plisky PJ, Rauh MJ, Kaminski TW, Underwood F. Star Excursion Balance Tests as a Predictor of Lower Extremity Injury in High School Basketball Players. J Orthop Sports Phys Ther. 2006;36(12):911-9.

23. Butler RJ, Lehr ME, Fink ML, Kiesel KB, Plisky PJ. Dynamic Balance Performance and Noncontact Lower Extremity Injury in College Football Players: An Initial Study. Sports Health: A Multidisciplinary Approach. 2013;5(5):417-22.

24. Redden J, Stokes K, Williams S. Establishing the Reliability and Limits of Meaningful Change of Lower Limb Strength and Power Measures during Seated Leg Press in Elite Soccer Players. J Sports Sci Med. 2018;17:539-46.

25. Reiser RF, Rocheford EC, Armstrong CJ. Building a better understanding of basic mechanical prinicples through analysis of the vertical jump. Strength & Conditioning Journal. 2006;28(4):70-80.

26. Maulder P, Cronin J. Horizontal and vertical jump assessment: reliability, symmetry, discriminative and predictive ability. Phys Ther Sport. 2005;6(2):74-82.

27. Slinde F, Suber C, Suber L, Edwen CE, Svantesson U. Test–Retest Reliability Of Three Different Countermovement Jumping Tests. J Strength Cond Res. 2008;22(2):640-3.

28. Hori N, Newton RU, Kawamori N, McGuigan MR, Kraemer WJ, Nosaka K. Reliability of performance measurements derived from ground reaction force data during countermovement jump and the influence of sampling frequency. Journal of Strength & Conditioning Research. 2009;23(3):874-82.
